# COVID-19 Genomic Surveillance in Bangui (Central African Republic) Reveals a Landscape of Circulating Variants Linked to Validated Antiviral Targets of SARS-CoV-2 Proteome

**DOI:** 10.3390/v15122309

**Published:** 2023-11-24

**Authors:** Ulrich Vickos, Marianna Camasta, Nicole Grandi, Sante Scognamiglio, Tobias Schindler, Marie Roseline Darnycka Belizaire, Ernest Lango-Yaya, Giscard Wilfried Koyaweda, Oscar Senzongo, Simon Pounguinza, Kaleb Kandou Jephté Francis Estimé, Stephanie N’yetobouko, Christelle Luce Bobossi Gadia, Dominos-Alfred Feiganazoui, Alain Le Faou, Massimiliano Orsini, Carlo Federico Perno, Luca Zinzula, Clotaire Donatien Rafaï

**Affiliations:** 1 Department of Diagnostic and Laboratory Medicine, UOC Microbiology and Immunology Diagnostics, Children’s Hospital Bambino Gesù, IRCCS, 00118 Rome, Italy; carlofederico.perno@opbg.net; 2 Department of Medicine, Infectious and Tropical Diseases, Sino-Central African Amitié Hospital, Bangui 94045, Central African Republic; 3 Laboratory of Molecular Virology, Department of Life and Environmental Sciences, University of Cagliari, 09042 Monserrato, Italy; marianna.camasta@unica.it (M.C.); sante.scognamiglio@unica.it (S.S.); 4 Department of Structural Molecular Biology, Max Planck Institute of Biochemistry, 82152 Martinsried, Germany; zinzula@biochem.mpg.de; 5 Swiss Tropical and Public Health Institute, 4123 Allschwil, Switzerland; tobias.schindler@swisstph.ch; 6 Department of Medical Parasitology and Infection Biology, University of Basel, 4051 Basel, Switzerland; 7 Central African Republic Office, World Health Organization (WHO) Representation, Bangui 94045, Central African Republic; belizairem@who.int; 8 Clinical Biology and Public Health National Laboratory, Bangui 94045, Central African Republic; elangoyaya@gmail.com (E.L.-Y.); gkoyaweda@gmail.com (G.W.K.); oscarsenzongo@yahoo.fr (O.S.); poungsims@gmail.com (S.P.); kalebkandou@gmail.com (K.K.J.F.E.); nyetoboukostephanie@yahoo.com (S.N.); bchristelleluce@yahoo.fr (C.L.B.G.); fedominosalfred@gmail.com (D.-A.F.); clotairerafai@yahoo.fr (C.D.R.); 9 EA 3452 CITHEFOR, Campus Brabois Santé, 54500 Vandœuvre-lès-Nancy, France; alainlefaou@gmail.com; 10 Faculty of Medicine, Maieutic and Health Sciences, University of Lorraine, Pole Brabois Santé, 54500 Nancy, France; 11 General and Experimental Microbiology, Laboratory of Microbial Ecology and Genomics of Microorganisms, Experimental Zooprophylactic Institute of the Venezie (IZSVe), 35020 Legnaro, Italy; morsini@izsvenezie.it

**Keywords:** Central African Republic, severe acute respiratory syndrome coronavirus 2, SARS-CoV-2, COVID-19, Delta variant, screening

## Abstract

Since its outbreak, Severe Acute Respiratory Syndrome Coronavirus 2 (SARS-CoV-2) spread rapidly, causing the Coronavirus Disease 19 (COVID-19) pandemic. Even with the vaccines’ administration, the virus continued to circulate due to inequal access to prevention and therapeutic measures in African countries. Information about COVID-19 in Africa has been limited and contradictory, and thus regional studies are important. On this premise, we conducted a genomic surveillance study about COVID-19 lineages circulating in Bangui, Central African Republic (CAR). We collected 2687 nasopharyngeal samples at four checkpoints in Bangui from 2 to 22 July 2021. Fifty-three samples tested positive for SARS-CoV-2, and viral genomes were sequenced to look for the presence of different viral strains. We performed phylogenetic analysis and described the lineage landscape of SARS-CoV-2 circulating in the CAR along 15 months of pandemics and in Africa during the study period, finding the Delta variant as the predominant Variant of Concern (VoC). The deduced aminoacidic sequences of structural and non-structural genes were determined and compared to reference and reported isolates from Africa. Despite the limited number of positive samples obtained, this study provides valuable information about COVID-19 evolution at the regional level and allows for a better understanding of SARS-CoV-2 circulation in the CAR.

## 1. Introduction

The Severe Acute Respiratory Syndrome Coronavirus 2 (SARS-CoV-2) emerged as a human pathogen in December 2019 in Wuhan, the capital of the Chinese Hubei Province, with an outbreak of severe pneumonia, the clinical manifestations of which took the name coronavirus disease 2019 (COVID-19) [1,2]. Thereafter, the virus spread worldwide as a pandemic and after more than three years has been responsible for 676 million cases and more than 6.8 million deaths [3]. Moreover, despite the availability of vaccines with more than thirteen billion doses administered so far, COVID-19 still poses a threat to global health. This is at least due in part to the lack of approved antiviral therapeutics administrable to non-hospitalized patients, as well as to the non-equitable access to COVID-19 vaccines among countries [4]. Furthermore, disparities in the application of, or adherence to, restriction policies, containment measures, diagnosis, and surveillance further exacerbate the risk of emergence of more virulent, transmissible, and vaccine-resistant SARS-CoV-2 variants [5]. Particularly, Africa was considered at high risk for COVID-19 spread due to the high number and diffusion of infectious diseases, inefficient health systems, and poverty, having only limited available resources to contain the virus [6]. In light of such a scenario, grim predictions had been made for the spread of COVID-19 across the African continent, although they were contradicted by relatively fewer cases in the continent as compared to the rest of the world, and with a lower case fatality ratio (CFR) during the first wave of the pandemic [7]. Indicators such as a younger population, comorbidity rates, urbanisation, population density, and poor virus detection capabilities have been proposed as drivers for this phenomenon, albeit with discrepancies in the impact of variables among countries [8]. During the second wave of infections, an overall increase in CFR was reported for all African Union member states, although disease severity and progression varied among them [9], thereby emphasizing the need to conduct studies that focus on regional levels for a better understanding of the impact of COVID-19 across Africa.

The Central African Republic (CAR) is located in the centre of the African continent, bordering Chad to the north, the Republic of Congo (ROC) and the Democratic Republic of Congo (DRC) to the south, Sudan and South Sudan to the east, and Cameroon to the west. Bangui, with an estimated population of 1,145,280 inhabitants in 2021, is the administrative capital and the main city of the CAR, with a multicultural mix resulting from being the main point of entry for expatriates and displaced people from central African territories [10]. Starting from the first case of COVID-19 reported on 14 March 2020, and referred to an immigrant patient, CAR has recorded, as of 2 July 2021, a total of 11,064 cumulative confirmed cases, of which 7142 were symptomatic (including 581 imported ones) and 3922 were asymptomatic, which were confirmed as resulting from local community transmission. Among 7103 patients treated, 98 died: 50 inside and 48 outside a hospital setting. In the main districts of the area selected for this study, i.e., Bangui, 78,685 people were vaccinated against COVID-19 [11]. Despite the spread of the virus in the country, the incidence of recorded cases was not as high as in countries like South Africa, Brazil, Italy, and the United States [12]. Most cases in the country have been reported in towns such as Bangui, Bouar, Berberati, and Mbaïki, but apparently with a slower spread since the lack of tests and the cost of analyses had led the CAR Ministry of Public Health to prioritize the testing of symptomatic subjects. These considerations suggest that official statistics are underestimated, as confirmed by a serological study that assessed the prevalence of antibodies (IgG Abs) against SARS-CoV-2 in 74.1% of the 799 asymptomatic participants [13].

As the virus spread and evolved, its genome accumulated many mutations identified in many of the 16,065,326 genome sequences submitted to GISAID (accessed on 2 October 2023) [14,15]. Interestingly, B.1.620 SARS-CoV-2 lineage was identified as originating specifically in the CAR, which brings mutations (like D614G on spike protein) and deletions already encountered in Variants of Concern (VoCs), increasing infectivity and resistance to the immune response [16]. Considered as a Variant Under Monitoring (VUM), B.1.620 lineage was also encountered in the Republic of Congo as one of the most common lineages circulating in the country [17]. However, after June 2021, B.1.620 was replaced by the Delta variant in the CAR [18].

Because of the lack of information, we performed a regional epidemiological investigation along with genomic surveillance in the CAR. We sampled 2687 individuals at the main entry points in the city of Bangui and the positive samples were selected for viral genome sequencing and determination of the mutations at the amino acid level.

## 2. Materials and Methods

### 2.1. Sampling Procedures

An epidemiological mass survey was conducted between 2 and 22 July 2021 to assess the spread of SARS-CoV-2 in the general population of Bangui at the main entry points of the city, which are Port-Beach, PK9 Bimbo, PK26, and LNBCSP. A total of 2687 participants were randomly included. Swabs were used to obtain the nasopharyngeal samples.

### 2.2. LumiraDx Antigen Detection Rapid Diagnostic Tests (Ag-RDT)

Samples were tested for SARS-CoV-2 antigens within hours using a microfluidic immunofluorescence assay on the LumiraDx™ platform (LumiraDx GmbH, Cologne, Germany). The tests were carried out according to the manufacturer’s protocol. Since the samples were taken on a viral transport medium (VTM), a mixture of equal volume of the VTM containing the swab and the diluent of the test on LumiraDx was prepared. This dilution rate was made according to the manufacturer’s recommendations and immediately tested on the instrument.

### 2.3. RT-PCR Test

All samples positive for the LumiraDx Test were retested using RT-PCR. Viral RNA was extracted from nasopharyngeal samples using the QIAamp Viral RNA Mini Kit (Qiagen, Hilden, Germany) according to the manufacturer’s instructions. A real-time PCR targeting SARS-CoV-2 ORF_1ab and N genes was performed using a specific PCR kit (SANSURE Biotech Kit, Changsha, People’s Republic of China) according to the manufacturer’s protocol using the 7500 Fast Dx Real-Time PCR Instrument (Applied Biosystems, Waltham, MA, USA).

### 2.4. Sample Selection and Sequencing

Positive samples for both antigen detection and RT-PCR test (CT value < 30) were retained for sequencing. These samples were sent to the National Research Institute of Kinshasa (DRC) for whole genome sequencing by Next Generation Sequencing (NGS).

### 2.5. NGS Sequencing

Libraries were prepared according to the COVID-19 ARTIC v3 Illumina Library Construction and Sequencing Protocol V.5 [19], using the NEBNext Ultra DNA Library Preparation Kit II (New England Biolabs Inc., Ipswich, MA, USA). Libraries were quantified (Qubit DNA BR, Thermo Scientific, Waltham, MA, USA), normalized, and pooled, and sequencing was performed using an Illumina MiSeq 100 (Illumina, San Diego, CA, USA).

### 2.6. Assembly

Raw reads were quality checked using the FastQC tool with default parameters [20]. Read quality was improved by read trimming using fastp software, version v0.23.4 [21]. Cleaned reads were assembled by reference-guided mapping using the Wuhan-1 reference sequence (accession ID: MN908947) and bowtie2 with default parameters [22]. A consensus sequence for the SARS-CoV-2 genome of each sample was obtained using the samtools/bcftool pipeline [23], and then polished by Pilon software, version 1.19 [24]. Sequences were annotated by searching for Open Reading Frames (ORFs) potentially encoding SARS-CoV-2 viral proteins as annotated in the Uniprot database [25]. Due to the low quality of assembled regions, ORFs showing > 50% of coverage were retained.

### 2.7. Phylogenetic Analyses and Variant/Lineage Identification

Alignments including low-coverage CAR sequences generated for this study (*n* = 29) as well as CAR and Africa sequences retrieved from GISAID and a set of reference sequences representing all main Variants of Concern (VoCs) from the NCBI database (Supplementary Table S1) were constructed using MAFFT v7 [26]. A Neighbour Joining (NJ) phylogenetic tree of CAR genomes was built using MEGA X software [27], using a p-distance model, and applying a pairwise deletion option. A maximum likelihood (ML) phylogenetic tree of African genomes was inferred using FastTree software, version 2.1.11 [28], which allows the computation of approximate ML trees for very large alignments, applying a generalized time-reversible (GTR) model of nucleotide evolution and CAT approximation for the varying rates of evolution across sites. Local support values were computed with the Shimodaira–Hasegawa test. Trees were visualized and analysed using FigTree v.1.4.4 [29]. For variant identification and mutation annotation, sequences were analysed using Nextclade [30].

### 2.8. Structural Analysis of SARS-CoV-2 Protein Mutants

Aminoacidic sequences of SARS-CoV-2 proteins were derived by analysing the Open Reading Frames (ORFs) from genome assembly via the online server tool ExPASy Translate (https://web.expasy.org/translate/, accessed on 14 November 2023) [31]. Reference sequences of the SARS-CoV-2 genome (NC_045512.2) and of the proteins NSP3 (YP_009725299.1) NSP5 (YP_009725301.1), NSP7 (YP_009725303.1), NSP8 (YP_009725304.1), NSP12 (YP_009725307.1), S (YP_009724390.1), and N (YP_009724397.2) were retrieved as FASTA format files from the NCBI Nucleotide and Protein databases, respectively. For each protein, the structural organization and amino acid positions corresponding to the boundaries of functional domains were defined according to the published literature and rendered as cartoon schemes onto which point mutations from the dataset were mapped on scale. Files for the 3D molecular viewing of experimentally solved protein structures available in Protein Data Bank (PDB) (NSP3: 7KAG (Ubl-1), 7JRN (Ubl-2-PLpro), 6Z5T (ADRP), 7LGO (NAB), 7RQG (Y3); NSP5: 6Y2E; NSP7/8/12 RdRp complex: 6M71; N: 7CDZ (NTD), 6YUN (CTD); S: 6VXX) were selected according to the highest resolution and earliest deposition criteria. Protein sequences were edited using PyMOL v.2.5.2 software [32] for the annotation of point mutations using the software mutagenesis tool, and protein structures were rendered as superimposed ribbon-backbone and isosurface representations shown at different angle orientations. Phylogenetic trees based on protein sequences were rendered using FigTree v.1.4.4 [29], and mutations identified within the dataset were mapped on the corresponding branches of each tree.

## 3. Results

### 3.1. Sampling, Processing, and Demographic Distribution

**Figure 1:**
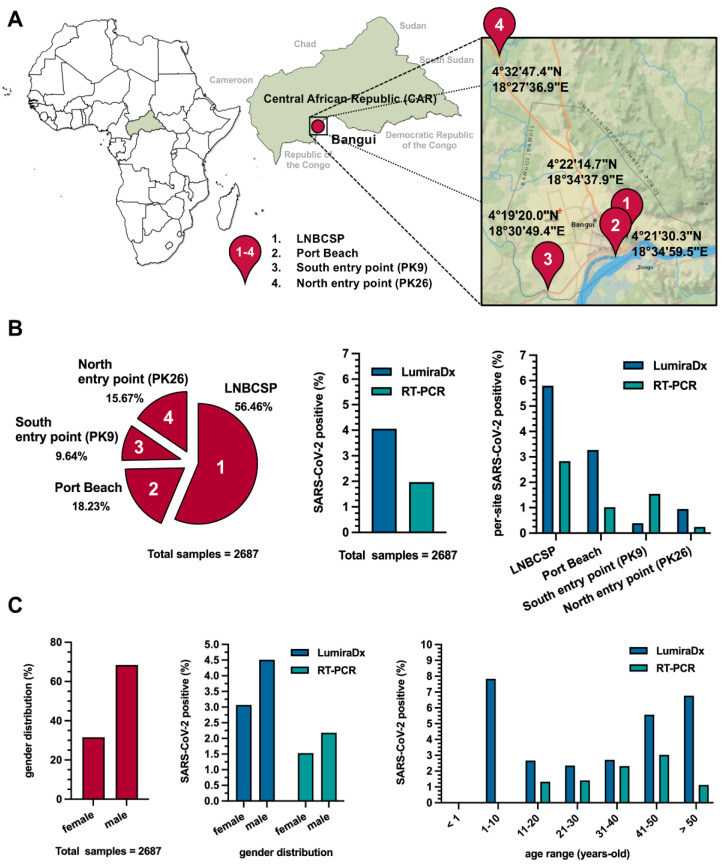
Geographic and demographic distribution of the samples. (**A**). Geographic distribution of the four different sampling checkpoints in Bangui, Central African Republic (CAR). (**B**). Sample distribution at the checkpoints (left); diverse percentage of subjects positive to Severe Acute Respiratory Syndrome Coronavirus 2 (SARS-CoV-2) depending on the test performed (middle) and depending on the collection point (right). (**C**). Demographic characteristics of the positive subjects compared to the totality of the participants: general gender distribution (left) and gender (middle) and age range (right) distribution according to the tests performed.

From April 2021, at the time of the second wave of the pandemic (*n* = 8294 confirmed cases and 79 deaths) to the beginning of July 2021, when we initiated this study, the number of cases increased rapidly, registering an amount of 11,075 confirmed cases and 98 deaths [33]. We carried out the SARS-CoV-2 screening study from 2 to 22 July 2021, with the enrolment of 2687 participants and the collection of their nasopharyngeal samples at the four main checkpoints of Bangui (Figure 1A). In total, 56.5% of the samples were collected at the Laboratoire National de Biologie Clinique et de Santé Publique (LNBCSP) from air-travelling passengers; 18.2% at Port Beach from travellers from North DRC; 15.7% at the North-PK26 entry point from road travellers and carriers from Cameroon, Nigeria, or south-west Chad, and the entry point; while 9.6% at the South-PK9 entry from participants coming from the border with the ROC and the CAR forest zone (Figure 1B). Of the 2687 samples tested, 109 (4.0%) resulted positive following the LumiraDx antigen test, and then only 53 (2.0%) were confirmed positive for SARS-CoV-2 by RT-PCR with a low Cq value. Among the latter, 13 were females and 40 males, all asymptomatic. The 31–50 years old age group was the most affected in the population, with an average age of 38 years (Figure 1C). A total of 29 of the 53 samples had a SARS-CoV-2 sequence available.

### 3.2. Phylogenetic Analysis

**Figure 2:**
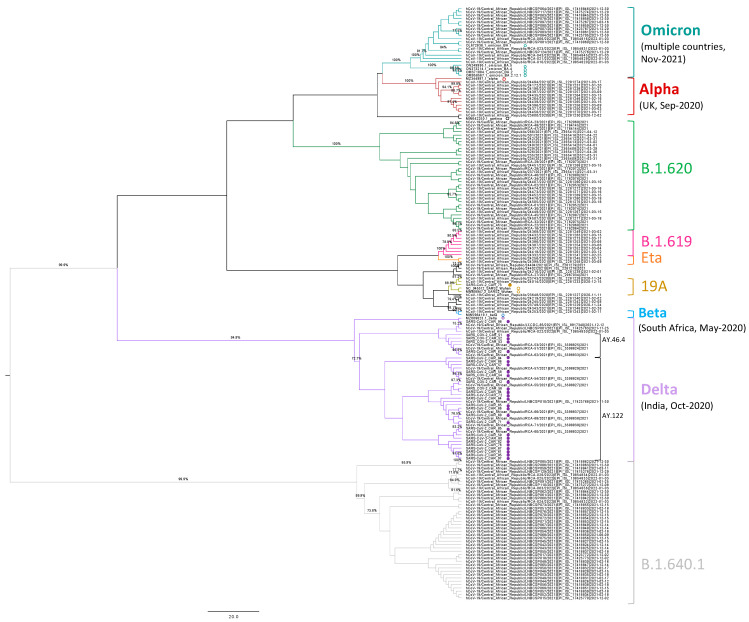
Phylogenetic tree of SARS-CoV-2 genomes circulating in the CAR. Neighbour Joining (NJ) phylogenetic tree of whole genome sequences of SARS-CoV-2 detected in the CAR, spanning from November 2020 to January 2022. Tree was built with MEGA X software. Bootstrap values ≥ 70 are shown on the branches. Pangolin clades and lineages are indicated with colours referred on the right side of the tree, with the Variants of Concern (VoCs) highlighted in bold. Reference sequences representing SARS-CoV-2 VoCs and CAR sequences from this study are labelled with an empty and a filled dot coloured according to the phylogenetic cluster they belong to, respectively. For VoCs, the date and country of the first report according to the World Health Organisation (WHO) are indicated between round brackets.

SARS-CoV-2 whole genome sequences from CAR samples collected between 2 and 22 July 2021 (*n* = 29) were compared in a Neighbour Joining (NJ) phylogenetic tree with the other sequences from CAR from the November 2020–January 2022 period, as retrieved from the GISAID database [15] (*n* = 138, low coverage excluded, listed in Supplementary Table S1) (Figure 2). Reference sequences for Wuhan wild type SARS-CoV-2 and the different VoCs (Supplementary Table S1) have also been included in the analysis. Both the SARS-CoV-2 genome sequences originated in this study and the ones retrieved from GISAID clustered together based on the lineage and clade, according to Nextclade analysis (which uses Pangolin lineage assignment) (Figure 2). The phylogenetic clusters identified in the CAR included one of the haplotypes originally circulating in Wuhan in December 2019 (lineage B, clade 19A, 88.8% bootstrap) and the VoCs Alpha (lineage B.1.1.7, clade 20I, 100% bootstrap), Beta (B.1.351, 20H, 90.2% bootstrap), Delta (lineage B.1.617.2 from clade 21A and lineages AY.46.4 and AY.122 from clade 21J), and Omicron from December 2021 (lineages BA.1, BA.1.1, BA.1.17.2, and BA.1.18 from clade 21K, 100% bootstrap) (Figure 2). The sequences from the present study (*n* = 29) clustered within the Delta clade, being split into the two subclusters of lineages AY.46.4 (*n* = 4) and AY.122 (*n* = 24, one clustered together with the Delta reference), except for one genome (SARS-CoV-2_CAR_73) that was associated with the 19A clade (Figure 2). Two additional major phylogenetic clusters comprised viral genomes from GISAID and were classified into lineages B.1.620 (*n* = 33, 100% bootstrap) and B.1.640.1 (*n* = 42, 99.9% bootstrap), both belonging to the 20A clade: the former circulated in the CAR between March and April 2021 while the latter spanned the whole year (Figure 2). Finally, a minority of sequences reported in the CAR were grouped into the Eta clade cluster (21D, lineage B.1.525, 100% bootstrap) and the B.1.619 lineage cluster (clade 20A, 100% bootstrap) (Figure 2). An additional maximum likelihood (ML) phylogenetic tree was built including our 29 sequences plus all the SARS-CoV-2 genomes, as reported in GISAID for Africa within the same period (2–22 July 2021, only high coverage, *n* = 2983, Supplementary Table S1) (Supplementary Figure S1). In line with CAR epidemiology, the analysis confirmed the extensive diffusion of the Delta VoC in African countries, representing 77% of all the reported sequences (*n* = 2311) and including different lineages from clades 21A, 21I, and 21J (Supplementary Figure S1). In addition, the African panorama appeared to be more heterogeneous, showing a wider diffusion of the Beta VoC that accounted for 19% of the genomes circulating in July 2021 (Supplementary Figure S1). The remaining 4% of sequences fell into clusters belonging to clades 19A and 19B (*n* = 6), 20A (*n* = 21), 20B (*n* = 17), 20C (*n* = 1), 20D (*n* = 42), 20I (Alpha VoC, *n* = 40), 21B (Kappa, *n* = 1), 21D (Eta, *n* = 2), and 21H (Mu, *n* = 2) (Supplementary Figure S1).

### 3.3. Structural Analysis

**Figure 3:**
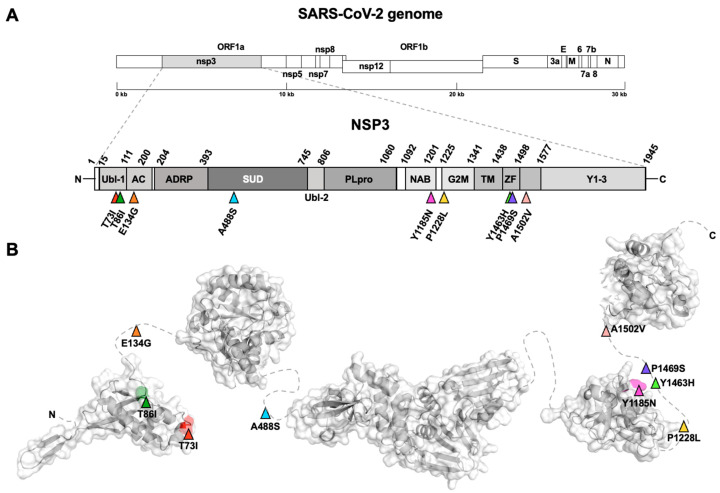
Non-structural proteins 3 (NSP3) schematic and tridimensional structure with reported mutations. (**A**). NSP3 structure scheme showing domain boundaries and non-synonymous mutations (coloured triangles). (**B**). NSP3 3D structure with the solved domains connected by dashed lines representing unsolved regions of the protein where most of the mutations found are indicated.

**Figure 4:**
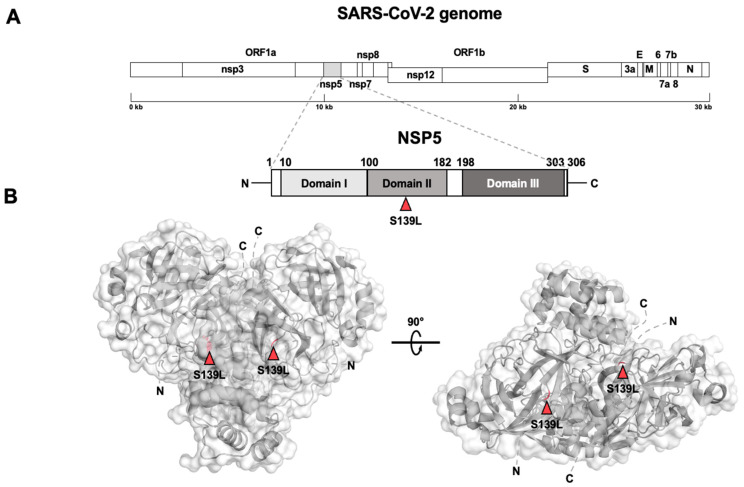
NSP5 schematic and tridimensional structure with reported mutations. (**A**) NSP5 structure scheme showing domain boundaries and the only one non-synonymous mutation found (in red). (**B**) NSP5 3D structure in the biological assembly as a dimer shown from different perspectives with the coloured mutation on each monomer.

The SARS-CoV-2 30kb genome encodes sixteen non-structural proteins (nsps) and four structural proteins (N, nucleocapsid, E, envelope, M, membrane, and S, spike). We decided to focus our analysis on the proteins validated as therapeutic targets, as NSP3 (multifunctional protein, including papain-like protease function), NSP5 (main protease), NSP8 (polymerase cofactor), NSP12 (RNA dependent RNA polymerase), and N and S protein. As an NSP12 cofactor, NSP7 was also included in the study, but mutations were absent in the respective aminoacidic sequence. We found nine mutations in the NSP3 sequence [34] (Figure 3A,B), specifically T73I and T86I in the ubiquitin-like domain 1 (Ubl-1), E134G in the Glu-rich acidic region (AC domain), A488S in the SARS-unique domain (SUD), Y1185N in the nucleic-acid-binding domain (NAB domain), P1228L in the *Betacoronavirus*-specific marker domain (G2M domain), and, finally, Y1463H, P1469S, and A1502V in the NSP3 ectodomain (3Ecto), also called zinc-finger domain (ZF domain). Among them, only A488S, P1228L, and P1469S mutations were reported as non-defining mutations for the Delta variant [35]. We located the single mutation S139L in the II 3C protease-like domain on NSP5 [36,37] (Figure 4A,B), present in only one of our samples (Supplementary Figure S2).

**Figure 5:**
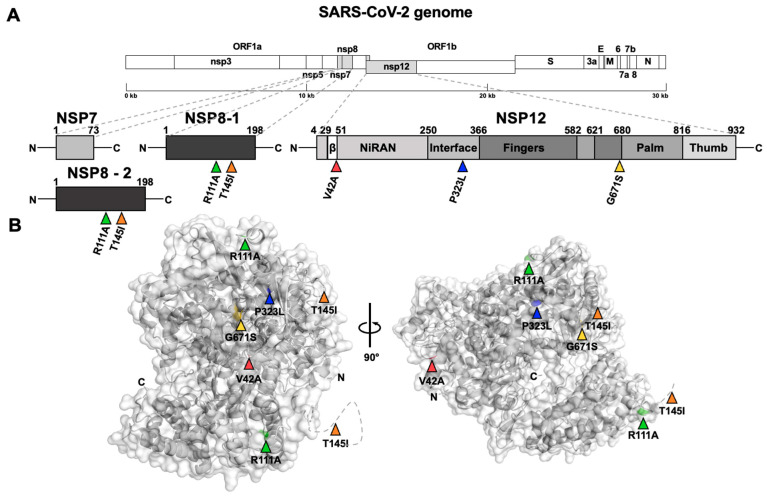
NSP12-7-8 schematic and tridimensional structure with reported mutations. (**A**) NSP12 and its cofactors NSP7 and NSP8 structure schemes showing domain boundaries and non-synonymous mutations as coloured triangles. (**B**) RNA dependent RNA polymerase (RdRp) complex (NSP12-NSP7-NSP8-NSP8) 3D structure in different orientations to show the mutations found.

We identified three mutations on NSP12 [38] (Figure 5A,B): V42A in the β-hairpin region, P323L on the interface domain, and G671S on the fingers’ subdomain. In addition, two mutations were found at the C-terminal domain of its cofactor NSP8: R111A and T145I. Particularly, P323L and G671S mutations presented a high frequency among the genomes we studied, as a confirmation of the presence of these mutations in the Delta variant [35], while V42A and the NSP8 mutations presented a very low frequency among our dataset (Supplementary Figure S3).

**Figure 6:**
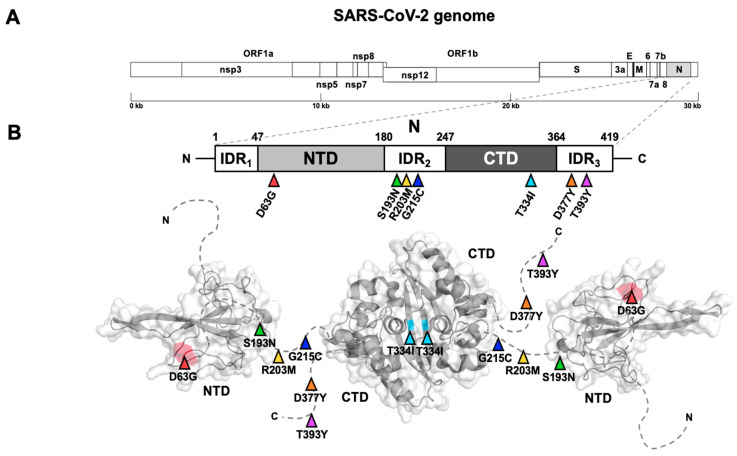
Nucleocapsid (N) schematic and tridimensional structure with reported mutations. (**A**) N structure scheme showing domain boundaries and non-synonymous mutations found as coloured triangles. (**B**) N 3D structure in the biological assembly as a dimer showing N-terminal Domain (NTD) and C-terminal Domain (CTD) solved portions with the coloured mutations on each monomer.

The N protein [39,40,41] showed seven mutations (Figure 6A,B): D63G in the N-terminal Domain (NTD); S193N, R203M, and G215C in the intrinsically disordered region (IDR) 1; T334I in the C-terminal domain (CTD); and D377Y and T393Y in the IDR2. We observed the same pattern, as for the other targets, with four of the seven mutations encountered in the Delta VoC, specifically D63G, R203M, and D377Y as defining mutations, and G215C as a non-defining mutation [35] (Supplementary Figure S4).

**Figure 7:**
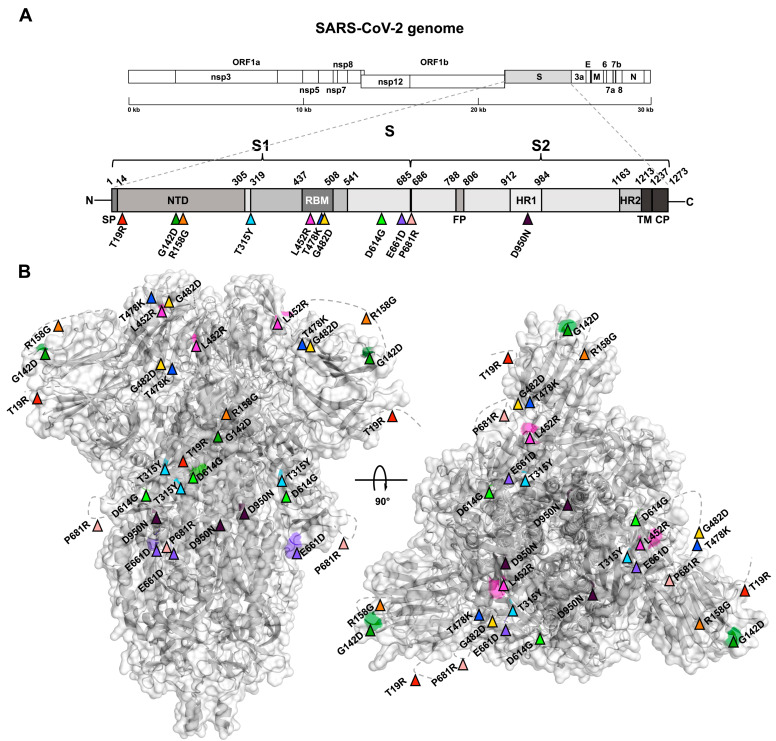
Spike protein (S) schematic and tridimensional structure with reported mutations. (**A**) S structure scheme showing domain boundaries and non-synonymous mutations as coloured triangles. (**B**) S 3D structure in its biological assembly as a trimer shown from two different perspectives with the coloured mutations on each monomer.

Among our targets, we identified the highest number of mutations on the spike protein (S) [42] (Figure 7A, B). On the one hand, ten mutations were located on the subunit S1, particularly T19R, G142D, and R158G in the N-terminal Domain (NTD); T315Y, D614G, E661D, and P681R in disordered regions; and L452R, T478K, and G482D in the Receptor Binding Domain (RBD). On the other hand, only the mutation D950N was located on the subunit S2 heptad repeat 1 (HR1). Except for T315Y, E661D, and G482D mutations that were present in a low frequency among our samples, all the others were classified as defining mutations for the Delta variant [35].

## 4. Discussion

To evaluate the presence of SARS-CoV-2 infection in the participants, we analysed the samples with two different techniques, the LumiraDx antigen test and the real-time PCR (RT-PCR). As expected, based on the different sensibility and specificity of the two techniques applied in attesting positive results, PCR was confirmed as the reference point-of-care (Figure 1).

The age of participants spanned from 1 to 50 years old, and the most affected age groups were 31–40 and 41–50 (Figure 1). Indeed, these age groups represent the most active part of the population because of their involvement in informal income-generating activities, and therefore their need to travel regularly.

In general, because of the very low number of positive samples in this study (circa 2%), we can state that their geographic distribution highlights a low circulation and spread of SARS-CoV-2 across the considered Bangui border checkpoints.

Overall, phylogenetic analyses showed that the Delta VoC had a widespread diffusion in the CAR as well as in other African countries, representing the great majority of sequences in our survey during July 2021 (Figure 2 and Supplementary Figure S1). While in the CAR these genomes were related mostly to the 21J clade, including lineages AY.46.4 and AY.122, the African scenario included 21A and 21I clades too. In addition, the Africa tree revealed a major presence of the Beta VoC, which was instead absent in the CAR in the same period. In line with its former presence in the CAR, the respective tree showed the abundant presence of B.1.620 (clade 20A): such a lineage was not represented in July 2021 since it was replaced by Delta VoC in the CAR after June 2021 [18]. Similarly, even if lineage B.1.640 (clade 20A) was first identified in France in April 2021 and was reported in the ROC since September 2021 [43], it was present in the CAR from January 2021 (Figure 2), likely suggesting earlier diffusion in the African continent. Finally, both analyses suggested the continuous residual circulation of the original Wuhan clades 19A and B (Figure 2 and Supplementary Figure S1).

Coherently with phylogenetic analyses, the mutations found in a high number of genomes from the dataset were mostly related to Delta VoC, both defining and non-defining the variant according to CoVariant [35]. Hence, their presence in the majority of sequences was expected. Contrarily, other mutations are not related to the variant widely circulating at the time of study and are present in only a few genomes, likely reflecting independent clusters of infection. An intermediate situation was found for NSP3, which harbours mutations that are not specifically linked to the Delta VoC, albeit showing a frequency similar to the ones indicated to be associated (as defining or non-defining) to this variant (Supplementary Figure S2). Among the Delta-associated mutations found in the different viral proteins, a number were reported to have an impact on viral fitness. For example, NSP12 mutations P323L and G671S were found to be associated with a higher activity and stability of the NSP7/8/12 complex [44]. R203M mutation in N protein plays a role in a higher spreading of the virus [45]. Likewise, many of the mutations found in the spike protein play key roles in helping the virus become more efficient in escaping the immune system. Particularly, T19R mutation in the NTD has been suggested to affect the binding of neutralizing antibodies [46], as well as L452R and T478K mutations in the RBD. More in detail, together with G142D and R158G mutations, T19R can avoid antibody binding helping variant transmission [47], while T478K can enhance ACE2 binding, as previously shown for D614G [48]. P681R mutation, at the boundary between S1 and S2 subunits, and D950N, on S2 subunit, can both increase the S1/S2 cleavage, enhancing viral replication [48,49]. Among the mutations not related to the Delta variant, only the E661D mutation has already been described elsewhere, as belonging to a Gamma-like-II lineage in the State of Paraná (Brazil) [50].

## 5. Conclusions

To date, only a few studies took into account SARS-CoV-2 diversity in Africa [18,51], and none of them were specifically focused on CAR. Therefore, despite the limited number of strains sequenced, regional studies like the present one could be highly informative. Indeed, both phylogenetic and structural analyses of the mutations found in the dataset showed that the major variant circulating in the CAR at the time of the study was the Delta one. Hence, enhanced epidemiological investigations and genomic characterization help to monitor viral evolution, detect new variants, elucidate transmission patterns, and identify clusters in outbreaks. Such actionable insights can be used to implement strategies to fight against the spread of SARS-CoV-2 variants, especially at borders and entry points. Admittedly, the first strain reported in the CAR was in an immigrant patient, identified thanks to the national strategy put in place for the detection of the various VoCs by the Ministry of Health (MoH) and its partners, mainly the World Health Organization (WHO). In this regard, this study may serve as a model for the better understanding and control of endemic disease circulation in African countries and advocate for the development of such study elsewhere in the continent. For this purpose, the accessibility of genomic tools (e.g., SARS-CoV-2 Africa dashboard for real-time COVID-19 information) [52] is essential to provide epidemiologic data about ongoing spreading events and to prevent future outbreaks of pathogens with epidemic potential, especially in areas with health management issues such as low-income countries.

## Data Availability

SARS-CoV-2 genome sequences generated in this study are available upon request.

## Supplementary Materials

The following supporting information can be downloaded at https://www.mdpi.com/article/10.3390/v15122309/s1, Figure S1: Africa tree; Figure S2: NSP3 and NSP5 trees; Figure S3: NSP12 and NSP8 trees; Figure S4: N and S trees; Table S1: List and accession numbers of publicly available sequences used in this study.
