# Supplementary material for: COVID-19 Genomic Surveillance in Bangui (Central African Republic) Reveals a Landscape of Circulating Variants Linked to Validated Antiviral Targets of SARS-CoV-2 Proteome
Source: Viruses. 2023 Nov 24;15(12):2309. doi: 10.3390/v15122309 (PMC10748234; doi:10.3390/v15122309)
Supplement: Supplementary file 1 [file viruses-15-02309-s001.zip › viruses-2674484-suppl.pdf]

viruses

MDPI

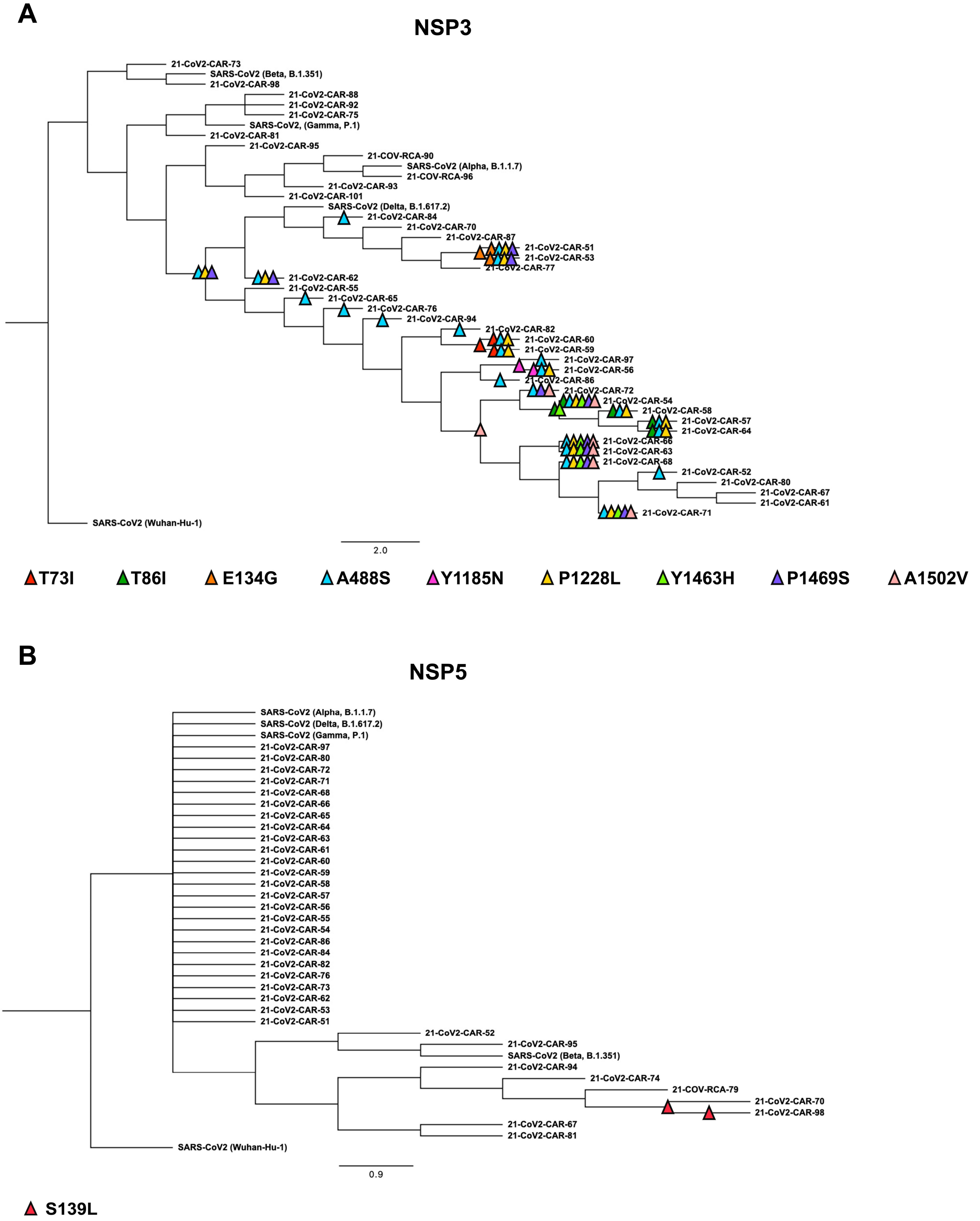

Figure S2. NSP3 and NSP5 trees. A. Phylogenetic trees of NSP3 with the legend reporting the mutations found in the dataset. Mutations are showed on the tree branches as coloured triangles. **B.** Phylogenetic trees of NSP5 with the legend reporting the single mutation found in the dataset. The mutation is showed on the tree branches as coloured triangles.

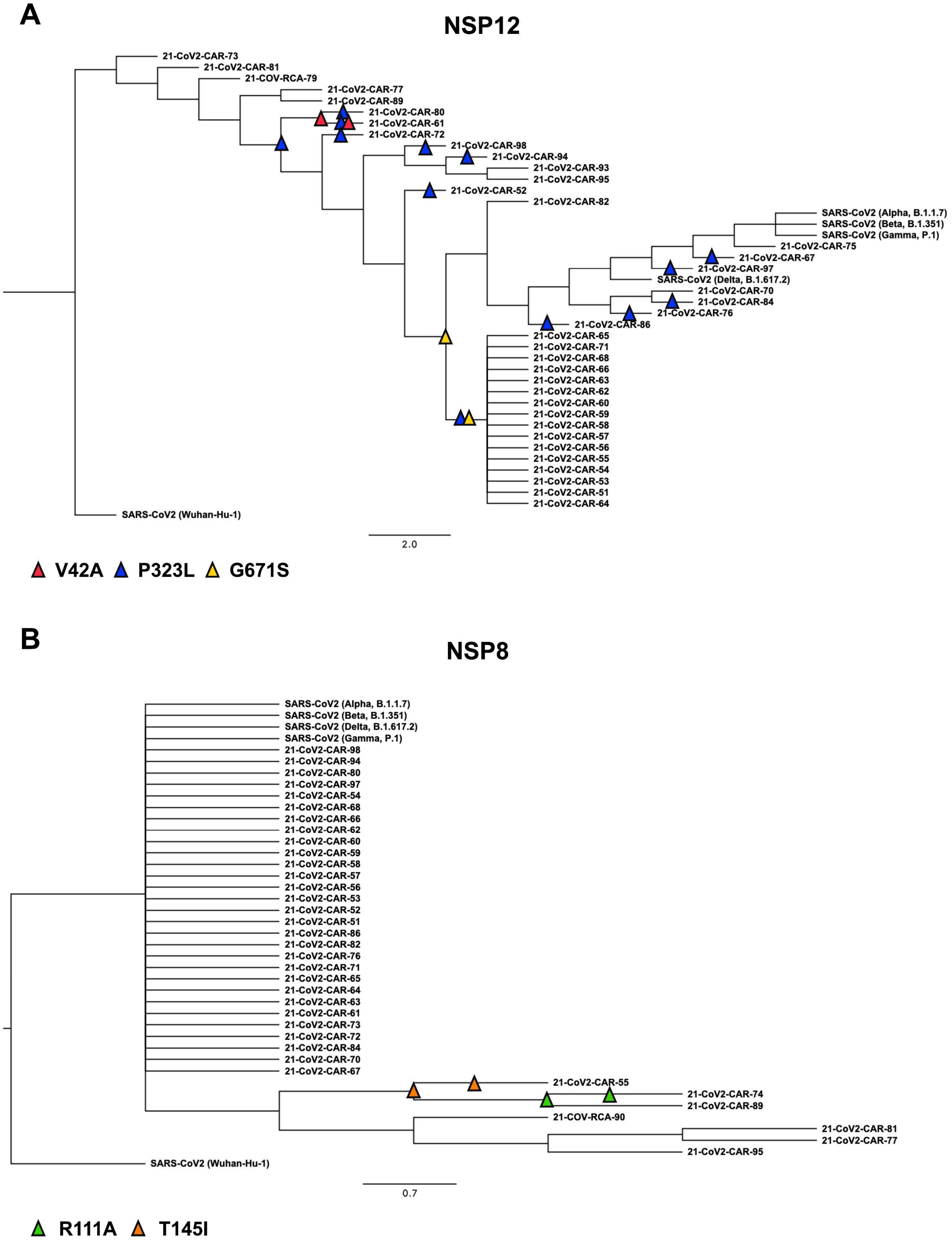

Figure S3. NSP12 and NSP8 trees. A. Phylogenetic trees of NSP12 with the legend reporting the mutations found in the dataset. Mutations are showed on the tree branches as coloured triangles. **B.** Phylogenetic trees of NSP8 with the legend reporting the mutations found in the dataset. Mutations are showed on the tree branches as coloured triangles.

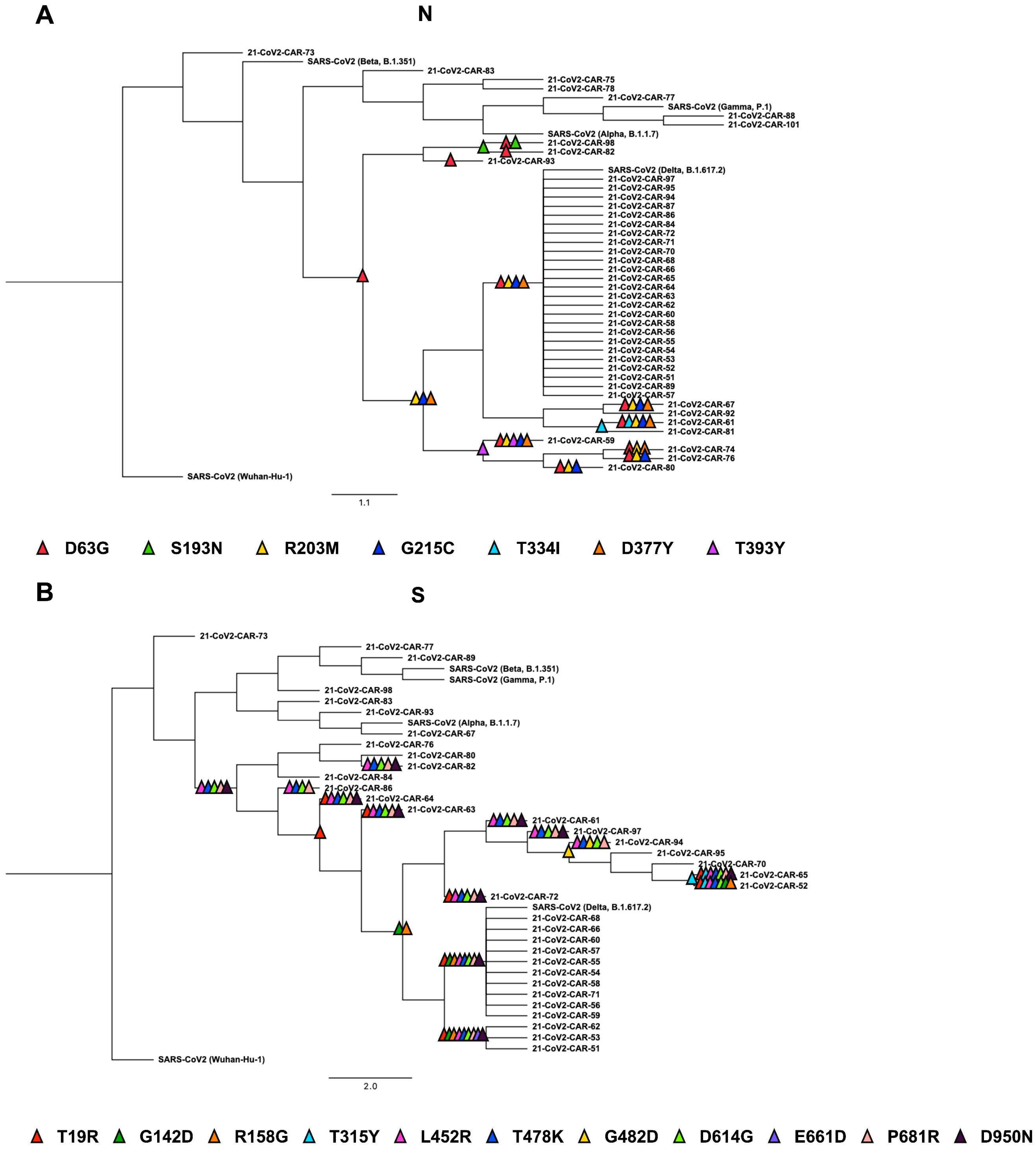

Figure S4. N and S trees. A. Phylogenetic trees of N with the legend reporting the mutations found in the dataset. Mutations are showed on the tree branches as coloured triangles. **B.** Phylogenetic trees of S with the legend reporting the mutations found in the dataset. Mutations are showed on the tree branches as coloured triangles.
