# Supplementary figures and images for: COVID-19 Genomic Surveillance in Bangui (Central African Republic) Reveals a Landscape of Circulating Variants Linked to Validated Antiviral Targets of SARS-CoV-2 Proteome
Source: Viruses. 2023 Nov 24;15(12):2309. doi: 10.3390/v15122309 (PMC10748234; doi:10.3390/v15122309)

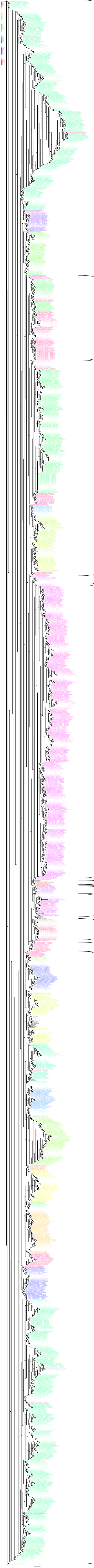

Delta – 21J

Delta – 21I

Delta – 21A

20A

Beta - 20H

Mu - 21H  
19A and 19B  
20A  
20B

Alpha - 20I

20D

Eta - 21D  
Delta - 21A

Delta –  
21J

Supplement: Supplementary file 1 [file viruses-15-02309-s001.zip › viruses-2674484-suppl-Figure S1.pdf]
